# Know Your Number; Control Your Number Campaign: Nigeria

**DOI:** 10.3389/fpubh.2026.1815662

**Published:** 2026-05-14

**Authors:** Nigeria Health Commissioners’ Forum (NHCF), Ayotunde Oguntade, Emmanuella Ezike, Ahmed Akorede Zakarriyah, Oladapo Awobeku

**Affiliations:** 1 Nigeria Health Commissioners Forum, Abuja, Nigeria; 2 Isuna Technologies, Abuja, Nigeria

**Keywords:** high blood glucose and blood pressure, Know Your Numbers, Nigeria, noncommunicable diseases, public health surveillance, screening

## Abstract

**Introduction:**

Know Your Numbers; Control Your Numbers is a landmark nationwide health initiative designed to screen 10 million Nigerians for risk factors associated with non-communicable diseases (NCDs), particularly high blood pressure and high blood glucose, implemented across all 36 states and the Federal Capital Territory.

**Methods:**

This policy paper presents insights from screening 1,551,394 individuals. Blood pressure was measured using the WHO HEARTS threshold (≥140/90 mmHg), and blood glucose was assessed using random testing (≥200 mg/dL). Measurements represent single-point screening findings and are not diagnostic.

**Results:**

Overall, 12% of participants had high blood pressure and 22% had high blood glucose. Both indicators rose with age: high blood pressure from 3.7% (18–29 years) to 23% (50+ years); high blood glucose from 20% to 24%. Males had marginally higher blood pressure (13% vs 12%), while females had higher blood glucose (23% vs 20%). Substantial geographic variation was observed: the North-West recorded the highest high blood pressure (15%) and the South-East the highest high blood glucose (47%). State-level rates ranged from 6.6% (FCT) to 19% (Plateau) for blood pressure, and from 1.4% (Nasarawa) to 50% (Delta) for blood glucose.

**Discussion:**

These findings provide an unprecedented national snapshot of NCD risk patterns. Extreme geographic variations may reflect demographic, methodological, or contextual factors and warrant further investigation. The campaign reinforces the urgent need for sustained, standardized screening, stronger integration of primary health care, and data-driven approaches to NCD prevention and control in Nigeria.

## Introduction

### Introduction to Know Your Number; Control Your numbers

The Know Your Numbers; Control Your Numbers campaign is a pivotal nationwide health campaign conceived to address the growing challenge of noncommunicable diseases (NCDs), particularly Hypertension and Diabetes, in Nigeria. Initiated and driven by the Nigeria Health Commissioners’ Forum, in partnership with Isuna, the ambitious project set out to screen 10 million Nigerians across all 36 states and the Federal Capital Territory (FCT). This initiative represents a significant national commitment to combating NCDs through proactive public health measures (1–3).

The project’s implementation across Nigeria was facilitated through collaboration between State Ministries of Health, State Primary healthcare development agencies, local government health authorities, and community leadership structures. The campaign strategically aligned with national health priorities and the specific health development plans of individual states, acknowledging the critical role of early detection and effective management in mitigating the morbidity and mortality associated with NCDs. The nationwide scope aimed to provide a comprehensive understanding of the NCD landscape, accounting for the diverse demographic, socioeconomic, and environmental contexts across the country (1, 3).

### Definition of key terms

#### High blood pressure (high blood pressure)

A systolic blood pressure ≥140 mmHg and/or diastolic blood pressure ≥90 mmHg measured on a single occasion, consistent with the WHO HEARTS threshold was used in this campaign (4). This does not constitute a clinical diagnosis of hypertension, which requires confirmation on at least two separate occasions.

#### Elevated blood glucose (high blood glucose)

A random blood glucose level ≥200 mg/dL (11.1 mmol/L), consistent with the American Diabetes Association threshold for a symptomatic provisional diagnosis (22). In the absence of fasting measurements or HbA1c confirmatory testing, these values are interpreted as screening-positive results and are not diagnostic of diabetes mellitus.

#### Noncommunicable diseases (NCDs)

Chronic diseases not transmitted from person to person, including cardiovascular diseases, cancers, chronic respiratory diseases, and diabetes, as defined by the World Health Organization (4).

#### Screening prevalence

The proportion of screened participants meeting a predefined threshold for a risk factor at a single point in time. This is distinct from population prevalence, which requires representative sampling and confirmatory testing.

#### Metabolic syndrome

A cluster of at least three of the following risk factors occurring simultaneously in an individual: central obesity, high blood pressure, elevated fasting glucose, elevated triglycerides, and reduced HDL cholesterol (5, 23).

### The burden of hypertension and diabetes in Nigeria

Noncommunicable diseases, with Hypertension and Diabetes at the forefront, have emerged as major public health concerns in Nigeria, contributing significantly to the national disease burden. According to estimates from the World Health Organization (WHO), NCDs are responsible for approximately 29% of all deaths in the country, with cardiovascular diseases being the predominant cause. National prevalence estimates for Hypertension varied, often cited between 22.5 and 28.9%, while diabetes affects an estimated 5.8% of the adult population. These figures highlight a substantial health challenge requiring concerted national action (3, 6, 16). Before the large-scale data collection facilitated by the Know Your Numbers; Control Your Numbers campaign, comprehensive, granular data on the burden and distribution of high blood pressure and elevated blood glucose across all states and geopolitical zones was often limited or fragmented. This initiative, therefore, plays a crucial role in establishing a robust baseline, identifying high-risk populations and regions, and providing the necessary evidence base for targeted interventions and informed policy development at both state and national levels.

### Objectives of the Know Your Numbers; Control Your Numbers campaign in Nigeria

The nationwide implementation of the Know Your Numbers initiative was guided by a set of interrelated objectives to establish a sustainable foundation for noncommunicable disease (NCD) prevention, early detection, and management in Nigeria. These objectives reflect the campaign’s dual focus on population-level awareness and system-level strengthening, aligning with the National Strategic Plan for NCD Prevention and Control (2023–2027) (14).

1. *Public awareness and health education*: To increase public understanding of high blood pressure and blood glucose as key risk factors for NCDs, while promoting knowledge of associated symptoms, complications, and preventive behaviors. The campaign aimed to tailor awareness messages to Nigeria’s diverse cultural and linguistic contexts to enhance community acceptance and engagement.
2. *Mass screening for NCD risk factors*: To conduct large-scale, accessible screening across all 36 states and the Federal Capital Territory (FCT), ensuring inclusion of both urban and rural populations. The goal was to identify individuals with elevated readings who might otherwise remain undiagnosed and unlinked to care.
3. *Early detection and referral strengthening*: To identify individuals presenting with high blood pressure and/or blood glucose and facilitate timely referral to appropriate health facilities for confirmatory testing, diagnosis, and continued management. This objective sought to strengthen the referral continuum within Nigeria’s healthcare system.
4. *Epidemiological data generation*: To systematically collect standardized, population-level data on the distribution and determinants of hypertension and diabetes. This data aimed to enhance Nigeria’s national health information system and provide a robust evidence base to inform NCD programming and policy formulation.
5. *Capacity building for health workers*: To strengthen the competencies of frontline health workers in NCD screening protocols, data recording, counseling, and basic management, thereby improving service delivery quality and reinforcing the resilience of the health system.
6. *Evidence-informed policy and planning*: To generate empirical insights capable of guiding national and state-level strategies for NCD prevention and control. The initiative sought to foster data-driven decision-making and to support the design of cost-effective, context-specific interventions aligned with Nigeria’s broader health priorities.

These objectives collectively aimed to build a strong foundation for long-term, sustainable NCD prevention and management across Nigeria, contributing to improved population health outcomes and reduced national disease burden.

## Methods

### Campaign strategy

The Know Your Numbers; Control Your Numbers campaign adopted a comprehensive, multi-pronged strategy across Nigeria to achieve its dual objectives of mass screening and nationwide awareness-raising about noncommunicable disease (NCD) risk factors. The implementation framework was deliberately designed to be adaptable across diverse state and community contexts while maintaining standardized national protocols.

- *Planning and coordination*: The campaign was coordinated through multi-tier structures at national, state, and local government levels. Implementation committees, comprising representatives from government health agencies, health facilities, civil society organizations, development partners, and community leaders, oversaw planning and execution. Strategic mapping guided the selection of screening locations based on population density, accessibility, and the presence of health facilities for effective referral and follow-up.
- *Community mobilization*: Community engagement was central to the campaign’s success. Awareness and mobilizations activities leveraged traditional, religious, and community leaders as champions, complemented by multi-lingual communication across radio, television, and social media. Community health workers and local organizations, including schools, women’s groups, and occupational associations, were instrumental in sensitizing residents, particularly in underserved and hard-to-reach areas. Community dialogs and outreach sessions were used to dispel myths, promote preventive behaviors, and encourage participation.
- *Participant identification and enrolment*: Participants were identified through a multi-channel community mobilization strategy. Community health workers, traditional and religious leaders, women’s groups, schools, and occupational associations were deployed across states to sensitize residents and encourage voluntary attendance at screening sites. Screening was conducted at both fixed health facilities and mobile outreach locations, with sites selected based on population density, accessibility, and proximity to referral facilities. All adults aged 18 years and above who presented at a designated screening point and provided verbal informed consent were eligible for participation. No formal sampling frame was applied; the campaign used an open-enrolment model to maximize reach. This approach, while enabling large-scale coverage, may have introduced self-selection bias, as discussed in the Limitations section (Table 1).

**Table 1:** Proportion of blood pressure and blood glucose.

Characteristic	*N* = 1,551,394*^1^*
Proportion of blood pressure	
BP_Status	
High	193,893 (12%)
Normal	1,357,500 (88%)
Unknown	1
Proportion of blood glucose	
BS_Status	
High	338,080 (22%)
Normal	1,213,313 (78%)
Unknown	1

*^1^n* (%).

### Screening implementation

Screening took place at both fixed health facilities and mobile outreach sites to ensure broad population coverage. The standardized screening protocol included:

- *Blood pressure measurement* using calibrated sphygmomanometers after a rest period of at least 5 min, following national and WHO HEARTS guidelines (threshold: ≥140/90 mmHg).
- *Blood glucose testing* using validated glucometers, primarily capturing random blood glucose levels (threshold: ≥200 mg/dL). No confirmatory fasting or HbA1c testing was performed, and thus, the findings are *not diagnostic* of diabetes.
- *Anthropometric assessments* (height, weight) and a brief health history covering lifestyle behaviors (smoking, alcohol use, physical activity) and medication use.

Participants with elevated readings were counseled immediately and referred to the nearest health facility for confirmatory assessment and management.

- *Quality assurance*: Quality control was embedded throughout the campaign. Standardized training sessions were conducted for all screening personnel to ensure accuracy and adherence to protocol. Equipment calibration, supervision by qualified health professionals, routine field monitoring, and data audits were instituted to minimize measurement and reporting errors. Feedback loops enabled continuous improvement and troubleshooting during implementation (Table 2).

**Table 2:** Proportion of blood pressure and blood glucose by state.

Characteristic	Proportion of blood pressure			Proportion of blood glucose		
	High *N* = 193,893*^1^*	Normal *N* = 1,357,500*^1^*	Overall *N* = 1,551,393*^1^*	High *N* = 338,080*^1^*	Normal *N* = 1,213,313*^1^*	Overall *N* = 1,551,393*^1^*
State						
Abia	10,780 (14%)	67,000 (86%)	77,780 (100%)	36,825 (47%)	40,955 (53%)	77,780 (100%)
Adamawa	2,152 (8.0%)	24,594 (92%)	26,746 (100%)	3,160 (12%)	23,586 (88%)	26,746 (100%)
Akwa Ibom	39 (17%)	192 (83%)	231 (100%)	34 (15%)	197 (85%)	231 (100%)
Anambra	51 (12%)	363 (88%)	414 (100%)	148 (36%)	266 (64%)	414 (100%)
Bauchi	14 (9.2%)	138 (91%)	152 (100%)	19 (13%)	133 (88%)	152 (100%)
Bayelsa	1,998 (18%)	8,874 (82%)	10,872 (100%)	1,136 (10%)	9,736 (90%)	10,872 (100%)
Benue	5,756 (8.9%)	59,270 (91%)	65,026 (100%)	2,754 (4.2%)	62,272 (96%)	65,026 (100%)
Borno	4,355 (10%)	37,387 (90%)	41,742 (100%)	3,448 (8.3%)	38,294 (92%)	41,742 (100%)
Cross River	30 (13%)	210 (88%)	240 (100%)	29 (12%)	211 (88%)	240 (100%)
Delta	11,656 (9.8%)	107,486 (90%)	119,142 (100%)	59,193 (50%)	59,949 (50%)	119,142 (100%)
Ebonyi	38 (13%)	251 (87%)	289 (100%)	74 (26%)	215 (74%)	289 (100%)
Edo	41 (13%)	276 (87%)	317 (100%)	58 (18%)	259 (82%)	317 (100%)
Ekiti	6,179 (8.7%)	65,130 (91%)	71,309 (100%)	27,918 (39%)	43,391 (61%)	71,309 (100%)
Enugu	33 (14%)	208 (86%)	241 (100%)	39 (16%)	202 (84%)	241 (100%)
FCT, Abuja	6,772 (6.6%)	96,095 (93%)	102,867 (100%)	9,098 (8.8%)	93,769 (91%)	102,867 (100%)
Gombe	655 (12%)	4,756 (88%)	5,411 (100%)	266 (4.9%)	5,145 (95%)	5,411 (100%)
Imo	517 (15%)	2,848 (85%)	3,365 (100%)	1,134 (34%)	2,231 (66%)	3,365 (100%)
Jigawa	18 (15%)	100 (85%)	118 (100%)	12 (10%)	106 (90%)	118 (100%)
Kaduna	10,522 (12%)	75,061 (88%)	85,583 (100%)	18,426 (22%)	67,157 (78%)	85,583 (100%)
Kano	52,792 (16%)	281,230 (84%)	334,022 (100%)	26,921 (8.1%)	307,101 (92%)	334,022 (100%)
Katsina	18 (13%)	120 (87%)	138 (100%)	34 (25%)	104 (75%)	138 (100%)
Kebbi	12 (15%)	69 (85%)	81 (100%)	11 (14%)	70 (86%)	81 (100%)
Kogi	7,362 (12%)	52,839 (88%)	60,201 (100%)	14,479 (24%)	45,722 (76%)	60,201 (100%)
Kwara	18,466 (18%)	84,044 (82%)	102,510 (100%)	29,370 (29%)	73,140 (71%)	102,510 (100%)
Lagos	4,989 (14%)	30,443 (86%)	35,432 (100%)	11,204 (32%)	24,228 (68%)	35,432 (100%)
Nasarawa	412 (15%)	2,382 (85%)	2,794 (100%)	39 (1.4%)	2,755 (99%)	2,794 (100%)
Niger	4,775 (12%)	34,856 (88%)	39,631 (100%)	4,264 (11%)	35,367 (89%)	39,631 (100%)
Ogun	22,852 (13%)	152,251 (87%)	175,103 (100%)	42,249 (24%)	132,854 (76%)	175,103 (100%)
Ondo	4,920 (9.6%)	46,415 (90%)	51,335 (100%)	12,767 (25%)	38,569 (75%)	51,336 (100%)
Osun	4,681 (8.0%)	53,725 (92%)	58,406 (100%)	27,017 (46%)	31,388 (54%)	58,405 (100%)
Oyo	119 (13%)	793 (87%)	912 (100%)	205 (22%)	707 (78%)	912 (100%)
Plateau	2,660 (19%)	11,699 (81%)	14,359 (100%)	2,610 (18%)	11,749 (82%)	14,359 (100%)
Rivers	5,750 (14%)	35,411 (86%)	41,161 (100%)	910 (2.2%)	40,251 (98%)	41,161 (100%)
Sokoto	11 (13%)	75 (87%)	86 (100%)	14 (16%)	72 (84%)	86 (100%)
Taraba	3 (7.1%)	39 (93%)	42 (100%)	7 (17%)	35 (83%)	42 (100%)
Yobe	2,459 (11%)	20,812 (89%)	23,271 (100%)	2,203 (9.5%)	21,068 (91%)	23,271 (100%)
Zamfara	6 (9.4%)	58 (91%)	64 (100%)	5 (7.8%)	59 (92%)	64 (100%)

^1^*n* (%).

### Data collection and management

Data collection and management across Nigeria relied on a robust system to handle the large volume of information generated by Know Your Numbers; Control Your Numbers, ensuring accuracy, confidentiality, and timely analysis.

#### Data collection tools

The data collection instrument was developed by the technical team at Isuna, in consultation with public health and epidemiology experts, to ensure comprehensiveness and contextual appropriateness. The tool was deployed as a web-based platform, accessible through tablets, smartphones, and computers, and is available here. The instrument captured a wide range of information, including demographic characteristics (age, sex, and geographic location), contact details, clinical screening results (blood pressure, random blood glucose), anthropometric measurements of behavioral risk factors (tobacco and alcohol use), family and medication history, and referral outcomes. To mitigate challenges in areas with limited connectivity, provisions were made for offline data entry with synchronization once internet access was restored, as well as paper-based back-ups.

All enumerators and state-level teams participated in structured training and familiarization sessions before field deployment. This training emphasized standardized use of the digital tool, data quality assurance protocols, and troubleshooting procedures, ensuring consistency and minimizing variability across data collection teams.

#### Data management process

Data management involves real-time or regular data entry, secure synchronization with central or state-level servers, implementation of data quality checks and validation procedures, safe storage that adhered to data protection principles, and systematic data cleaning and preparation for analysis.

### Data analysis

Data analysis was conducted using R statistical software (version 4.4.1, 2024 release). Descriptive statistics (means, proportions, and prevalence estimates) were used to summarize demographic and clinical characteristics. Cross-tabulations were conducted to explore variations by age, sex, state, and geopolitical zone. Inferential analyses, including chi-square tests, were applied to examine the associations between demographic and behavioral risk factors and clinical outcomes, with statistical significance assessed using *p*-values. The analysis focused on estimating the proportion of high blood glucose and high blood pressure, identifying patterns of distribution across sub-populations, and exploring associations with behavioral and demographic characteristics as well as medication use patterns. Findings were intended to provide a robust evidence base to inform health policy and program implementation (1, 7).

Although body mass index (BMI) was calculated from collected anthropometric data (height and weight), its association with high blood pressure and blood glucose was not examined in this analysis. Future analysis should incorporate BMI as a continuous or categorical variable to examine its independent contribution to NCD risk within this population.

### Ethical considerations

Participation in the Know Your Numbers; Control Your Numbers campaign was voluntary, with informed consent obtained from all participants before screening. Personal identifiers were collected only for referral purposes and handled confidentially in line with the Nigeria Data Protection Regulation (24) and ethical guidance from the Federal Ministry of Health.

All health workers were trained on participant rights, confidentiality, and ethical conduct. Individuals with elevated readings received on-site counseling and referrals for confirmatory testing. The Nigeria Health Commissioners’ Forum provided ethical oversight in accordance with the National Health Research Ethics Code.

## Results and discussion

This section presents the key findings from the Know Your Numbers; Control Your Numbers campaign, which generated one of the most extensive datasets on noncommunicable disease (NCD) risk factors in Nigeria. The results highlight national trends and subnational variations in high blood pressure and blood glucose, alongside demographic, behavioral, and geographic patterns observed among more than 1.5 million screened individuals. While the findings offer valuable insights into the distribution of NCD risks, they are based on single-point screening data. They should be interpreted as indicative rather than diagnostic of hypertension and diabetes.

### Screening outcomes and key findings

**Figure 1:**
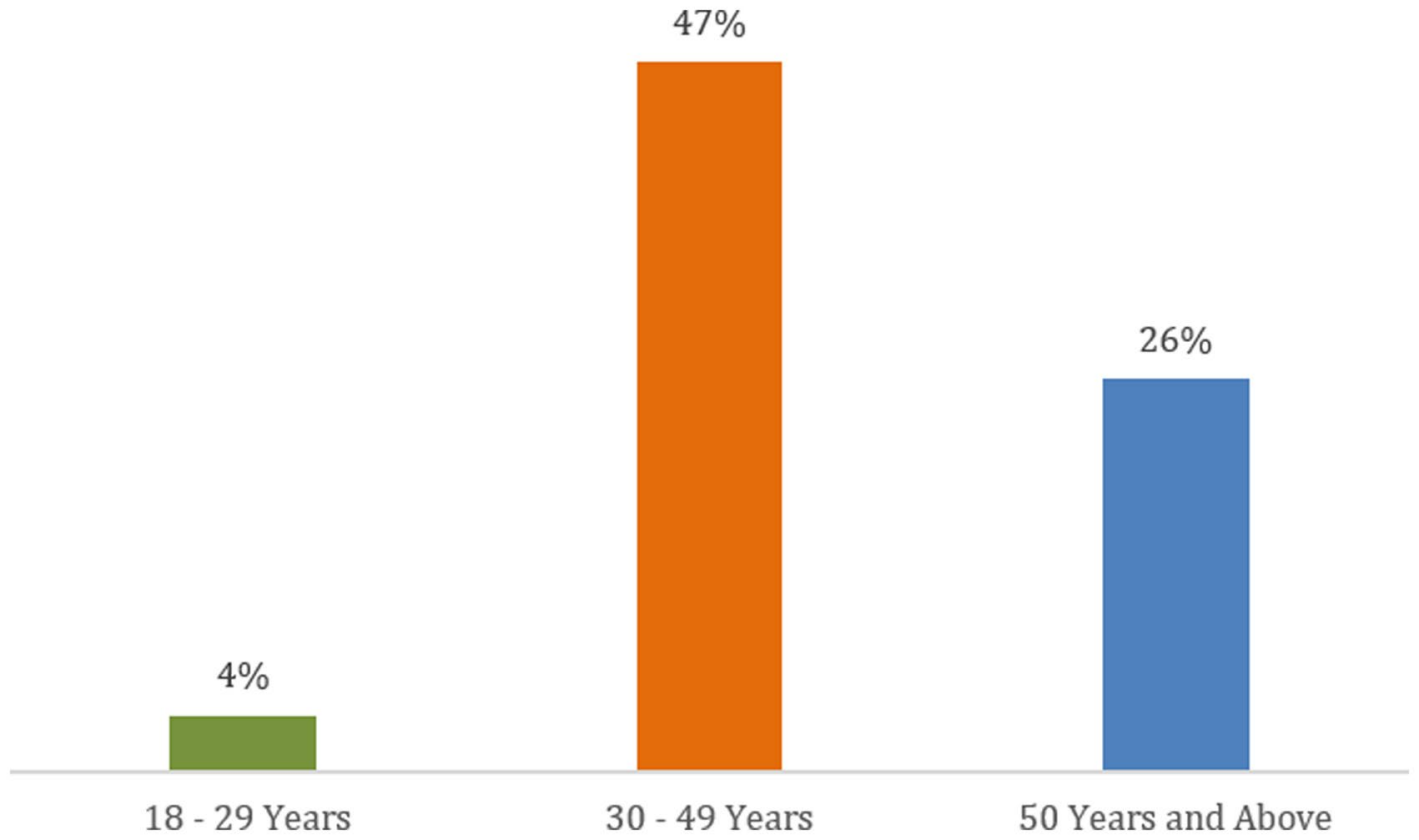
Age distribution of screened participants *N* = 1,551,393.

**Figure 2:**
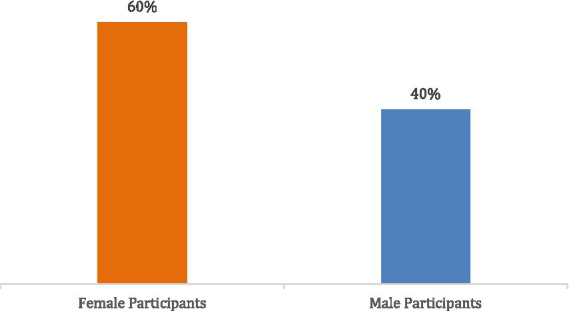
Gender distribution of screened participants *N* = 1,551,393.

1. *Number of people screened*: Know Your Numbers; Control Your Numbers successfully screened 1,551,393 individuals across participating states in Nigeria. This large sample size provides a robust foundation for understanding of the distribution patterns of high blood pressure and high blood glucose across the Nation, as a foundation for further research, follow-up and diagnosis.
2. *Demographic characteristics of study participants*: The screened population encompassed a diverse range of Nigerians:

◦ *Age distribution:* The participants included 414,102 individuals (27%) aged 18–29 years, 731,145 individuals (47%) aged 30–49 years, and 406,146 individuals (26%) aged 50 years and above, indicating substantial representation across adult age groups (Figure 1).
◦ *Gender distribution:* The sample comprised 928,786 females (60%) and 622,607 males (40%), indicating a higher participation rate among females (Figure 2).
◦ *Geographic distribution:* Participants were drawn from all six geopolitical zones, providing a basis for regional comparisons. The South-West zone had the highest number of participants (392,497), while the South-East had the lowest (82,089), although proportion data accounts for these differences.

**Figure 3:**
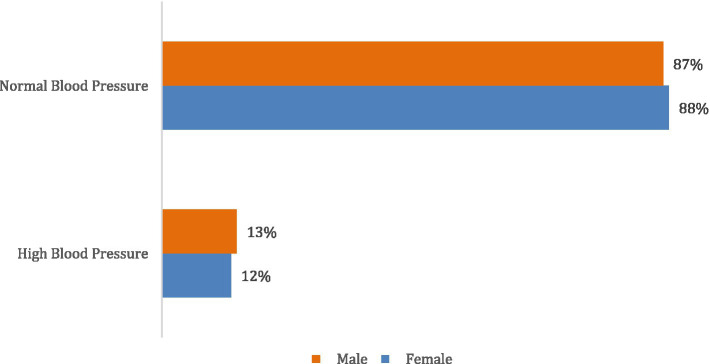
Blood pressure readings of screened participants categorized by gender.

**Figure 4:**
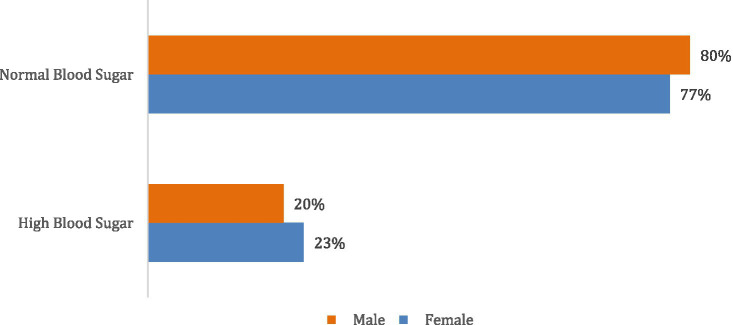
Blood glucose readings of screened participants categorized by gender.

3. *Current health status: blood pressure and blood glucose*: At the time of screening:

◦ *High blood pressure* was recorded in 12% of participants (*n* = 193,893), defined as systolic ≥140 mmHg and/or diastolic ≥90 mmHg or self-reported use of antihypertensive medication (Tables 3–6).
◦ *High blood glucose* was detected in 22% of participants (*n* = 338,080), defined as random blood glucose ≥200 mg/dL or self-reported use of glucose-lowering medication (Figure 3; Figure 4).

**Table 3:** Proportion of blood pressure and blood glucose by socio-demographics.

Characteristic	Proportion of blood pressure			Proportion of blood glucose		
	High *N* = 193,893*^1^*	Normal *N* = 1,357,500*^1^*	Overall *N* = 1,551,393*^1^*	High *N* = 338,080*^1^*	Normal *N* = 1,213,313*^1^*	Overall *N* = 1,551,393*^1^*
Age_group						
18–29_years	15,190 (3.7%)	398,912 (96%)	414,102 (100%)	81,807 (20%)	332,294 (80%)	414,101 (100%)
30–49_years	85,040 (12%)	646,105 (88%)	731,145 (100%)	158,214 (22%)	572,931 (78%)	731,145 (100%)
50 + _years	93,663 (23%)	312,483 (77%)	406,146 (100%)	98,059 (24%)	308,088 (76%)	406,147 (100%)
Gender						
Female	114,023 (12%)	814,763 (88%)	928,786 (100%)	211,550 (23%)	717,237 (77%)	928,787 (100%)
Male	79,870 (13%)	542,737 (87%)	622,607 (100%)	126,530 (20%)	496,076 (80%)	622,606 (100%)
Geopolitical_Zone						
North-Central	46,203 (12%)	341,185 (88%)	387,388 (100%)	62,614 (16%)	324,774 (84%)	387,388 (100%)
North-East	9,638 (9.9%)	87,726 (90%)	97,364 (100%)	9,103 (9.3%)	88,261 (91%)	97,364 (100%)
North-West	63,379 (15%)	356,713 (85%)	420,092 (100%)	45,423 (11%)	374,669 (89%)	420,092 (100%)
South-East	11,419 (14%)	70,670 (86%)	82,089 (100%)	38,220 (47%)	43,869 (53%)	82,089 (100%)
South–South	19,514 (11%)	152,449 (89%)	171,963 (100%)	61,360 (36%)	110,603 (64%)	171,963 (100%)
South-West	43,740 (11%)	348,757 (89%)	392,497 (100%)	121,360 (31%)	271,137 (69%)	392,497 (100%)

^1^*n* (%).

**Table 4:** Cross-tabulation of blood pressure and blood glucose by socio-demography.

Characteristic	Blood pressure				Blood glucose			
	Overall *N* = 1,551,393*^1^*	High *N* = 193,893*^1^*	Normal *N* = 1,357,500*^1^*	*p*-value*^2^*	Overall *N* = 1,551,393*^1^*	High *N* = 338,080*^1^*	Normal *N* = 1,213,313*^1^*	*p*-value*^2^*
Age_group				<0.001				<0.001
18–29_years	414,102 (100%)	15,190 (3.7%)	398,912 (96%)		414,101 (100%)	81,807 (20%)	332,294 (80%)	
30–49_years	731,145 (100%)	85,040 (12%)	646,105 (88%)		731,145 (100%)	158,214 (22%)	572,931 (78%)	
50 + _years	406,146 (100%)	93,663 (23%)	312,483 (77%)		406,147 (100%)	98,059 (24%)	308,088 (76%)	
Gender				**<0.001**				**<0.001**
Female	928,786 (100%)	114,023 (12%)	814,763 (88%)		928,787 (100%)	211,550 (23%)	717,237 (77%)	
Male	622,607 (100%)	79,870 (13%)	542,737 (87%)		622,606 (100%)	126,530 (20%)	496,076 (80%)	
Smoke				**<0.001**				0.13
Ever Smoked	58,545 (100%)	8,522 (15%)	50,023 (85%)		58,545 (100%)	12,907 (22%)	45,638 (78%)	
Never Smoked	1,492,848 (100%)	185,371 (12%)	1,307,477 (88%)		1,492,848 (100%)	325,173 (22%)	1,167,675 (78%)	
Geopolitical_Zone				**<0.001**				**<0.001**
North-Central	387,388 (100%)	46,203 (12%)	341,185 (88%)		387,388 (100%)	62,614 (16%)	324,774 (84%)	
North-East	97,364 (100%)	9,638 (9.9%)	87,726 (90%)		97,364 (100%)	9,103 (9.3%)	88,261 (91%)	
North-West	420,092 (100%)	63,379 (15%)	356,713 (85%)		420,092 (100%)	45,423 (11%)	374,669 (89%)	
South-East	82,089 (100%)	11,419 (14%)	70,670 (86%)		82,089 (100%)	38,220 (47%)	43,869 (53%)	
South–South	171,963 (100%)	19,514 (11%)	152,449 (89%)		171,963 (100%)	61,360 (36%)	110,603 (64%)	
South-West	392,497 (100%)	43,740 (11%)	348,757 (89%)		392,497 (100%)	121,360 (31%)	271,137 (69%)	

^1^*n* (%). ^2^Pearson’s Chi-squared test. Bold values indicate statistically significant associations (*p* < 0.001) based on Pearson’s Chi-squared test.

**Table 5:** Cross-tabulation of blood pressure and blood glucose by lifestyle factors.

Characteristic	Blood pressure				Blood glucose			
	Overall *N* = 1,551,393*^1^*	High *N* = 193,893*^1^*	Normal *N* = 1,357,500*^1^*	*p*-value*^2^*	Overall *N* = 1,551,393*^1^*	High *N* = 338,080*^1^*	Normal *N* = 1,213,313*^1^*	*p*-value*^2^*
Smoke				**<0.001**				0.13
Ever Smoked	58,545 (100%)	8,522 (15%)	50,023 (85%)		58,545 (100%)	12,907 (22%)	45,638 (78%)	
Never Smoked	1,492,848 (100%)	185,371 (12%)	1,307,477 (88%)		1,492,848 (100%)	325,173 (22%)	1,167,675 (78%)	
Alcohol				**<0.001**				**<0.001**
Ever Taken Alcohol	209,140 (100%)	27,994 (13%)	181,146 (87%)		209,140 (100%)	59,654 (29%)	149,486 (71%)	
Never Taken Alcohol	1,342,253 (100%)	165,899 (12%)	1,176,354 (88%)		1,342,253 (100%)	278,426 (21%)	1,063,827 (79%)	
Exercise				**0.007**				**<0.001**
Ever exercise	973,381 (100%)	121,113 (12%)	852,268 (88%)		973,381 (100%)	229,574 (24%)	743,807 (76%)	
Never exercise	578,012 (100%)	72,780 (13%)	505,232 (87%)		578,012 (100%)	108,506 (19%)	469,506 (81%)	

^1^*n* (%). ^2^Pearson’s Chi-squared test. Bold values indicate statistically significant associations (*p* < 0.001) based on Pearson’s Chi-squared test.

**Table 6:** Cross-tabulation of blood pressure and blood glucose medication use.

Characteristic	Overall *N* = 210,054*^1^*	High *N* = 42,350*^1^*	Normal *N* = 167,704*^1^*	*p*-value*^2^*
Are you currently on medication for Hypertension				<0.001
*No*	61,380 (100%)	12,791 (21%)	48,589 (79%)	
*Yes*	148,674 (100%)	29,559 (20%)	119,115 (80%)	

Characteristic	Overall *N* = 88,615*^1^*	High *N* = 19,838*^1^*	Normal *N* = 68,777*^1^*	*p*-value*^2^*
Currently on medication fordiabetess				0.030
No	34,389 (39%)	7,567 (38%)	26,822 (39%)	
Yes	54,226 (61%)	12,271 (62%)	41,955 (61%)	

^1^*n* (%). ^2^Pearson’s Chi-squared test.

These findings indicate a substantial burden of NCD risk across the screened population. However, because the measurements were taken at a single time point and were not confirmed by fasting glucose or repeat blood pressure readings, they should not be interpreted as clinical prevalence estimates.

**Figure 5:**
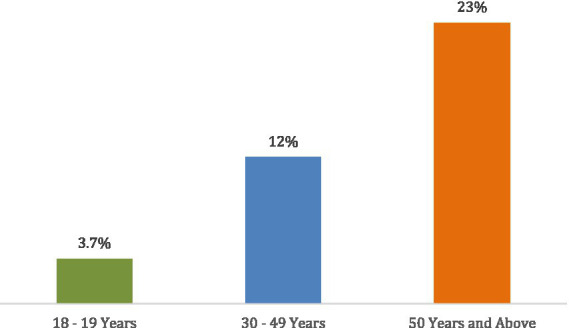
Proportion of high blood pressure in age-related patterns.

**Figure 6:**
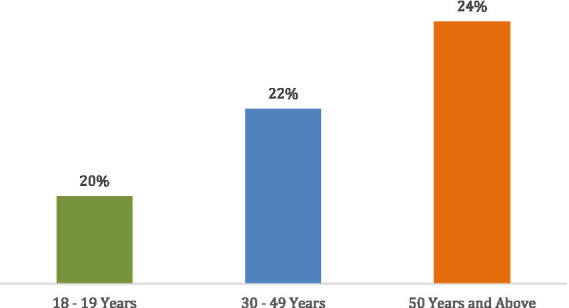
Proportion of high blood glucose in age-related patterns.

**Figure 7:**
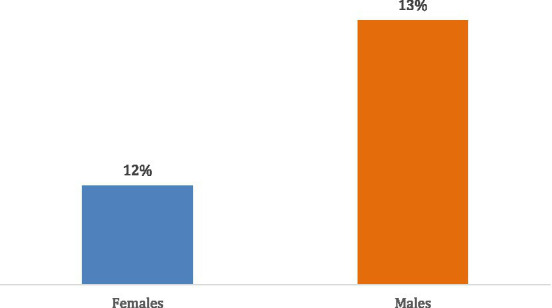
Prevalence of high blood pressure in gender-related patterns.

**Figure 8:**
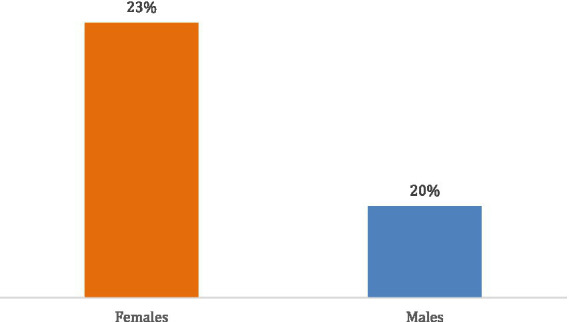
Prevalence of high blood glucose in gender-related patterns.

4. Burden by age groups and gender:

◦ *Age-related patterns*: The likelihood of high blood pressure increased sharply with age, from 3.7% among those aged 18–29 years to 12% among those aged 30–49 years and 23% among participants aged 50 and above (*p* < 0.001). High blood glucose followed a similar though less pronounced pattern, rising from 20 to 24% across these age groups (Figure 5; Figure 6).
◦ *Gender-related patterns*: Males had a slightly higher proportion of high blood pressure readings (13%) than females (12%), while females had a higher proportion of high blood glucose (23%) than males (20%). These trends may reflect both biological differences and behavioral factors such as dietary patterns, care-seeking behavior, and physical activity levels (Figure 7; Figure 8).

5. *Geographic distribution of NCDs*: Substantial regional and state-level variations were observed across Nigeria (18):

◦ *High Blood Pressure:* ranged from 9.9% in the North-East to 15% in the North-West. State-level variations were considerable, with rates as high as 18% in Bayelsa and Kwara, and 19% in Plateau, contrasting with lower rates such as 6.6% in FCT and 8.0% in Adamawa and Osun.
◦ *High blood glucose:* varied even more widely from 8.8% in FCT and 9.3% in the North-East to 47% in the South-East. State highlights include Abia (47%), Delta (50%), and Osun (46%) with very high rates, compared to extremely low rates in Benue (4.2%), Gombe (4.9%), Nasarawa (1.4%), and Rivers (2.2%). These dramatic variations necessitate an investigation into underlying factors and the development of highly localized intervention strategies.

#### State-wise insight of high blood pressure

Associations between lifestyle behaviors and screening outcomes revealed patterns largely consistent with existing evidence, though some findings warrant deeper investigation. The rates varied widely, indicating diverse levels of burden across the country.

**Figure 9:**
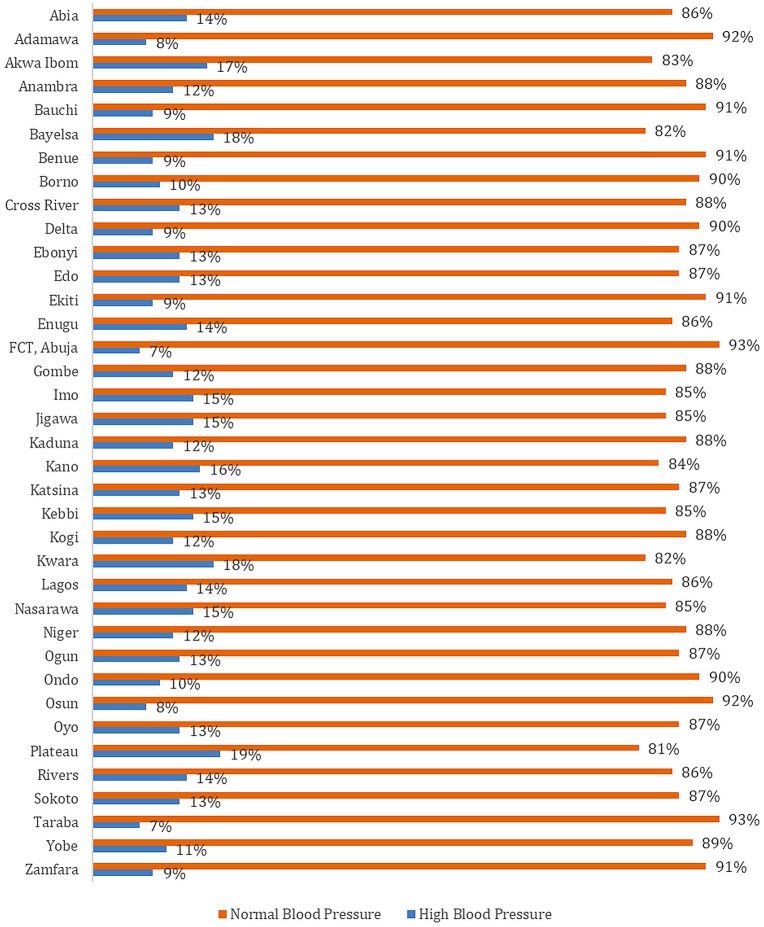
Blood pressure readings of screened participants categorized by state.

- *Highest burden states*: The state-level distribution of high blood pressure was notably heterogeneous (Figure 9). The threefold difference between the highest-burden states (Plateau, Bayelsa, Kwara) and the lowest (FCT, Taraba, Adamawa) warrants investigation into whether these gradients reflect genuine epidemiological variation or methodological inconsistencies such as device calibration differences or sampling disparities.
- *Moderate burden states*: Many states fell within the moderate range, typically between 10 and 15%. Examples include Lagos (14%), Abia (14%), Imo (15%), Jigawa (15%), Kebbi (15%), and Nasarawa (15%).
- *Lowest burden states*: The Federal Capital Territory (FCT), Abuja, had the lowest recorded readings at 6.6%. Taraba (7.1%), Adamawa (8.0%), Osun (8.0%) (19), Ekiti (8.7%), and Benue (8.9%) also demonstrated rates below 9%.

The observed range highlights that the risk or burden of High Blood Pressure is not uniform across Nigeria, with some states facing nearly three times the rates of others.

#### State-wise insight of high blood glucose

The disparities in the burden of high blood glucose across states were even more dramatic than those observed for High Blood Pressure.

*Highest burden states*: Delta reported the highest reading at 50%, followed by Abia (47%) and Osun (46%). Ekiti (39%), Anambra (36%), and Imo (34%) also showed very high rates.

*Moderate burden states*: States like Kaduna (22%), Oyo (22%), Kogi (24%), Ogun (24%), Ondo (25%), Katsina (25%), Ebonyi (26%), and Kwara (29%) fell into a broad moderate category, though still representing a significant burden.

**Figure 10:**
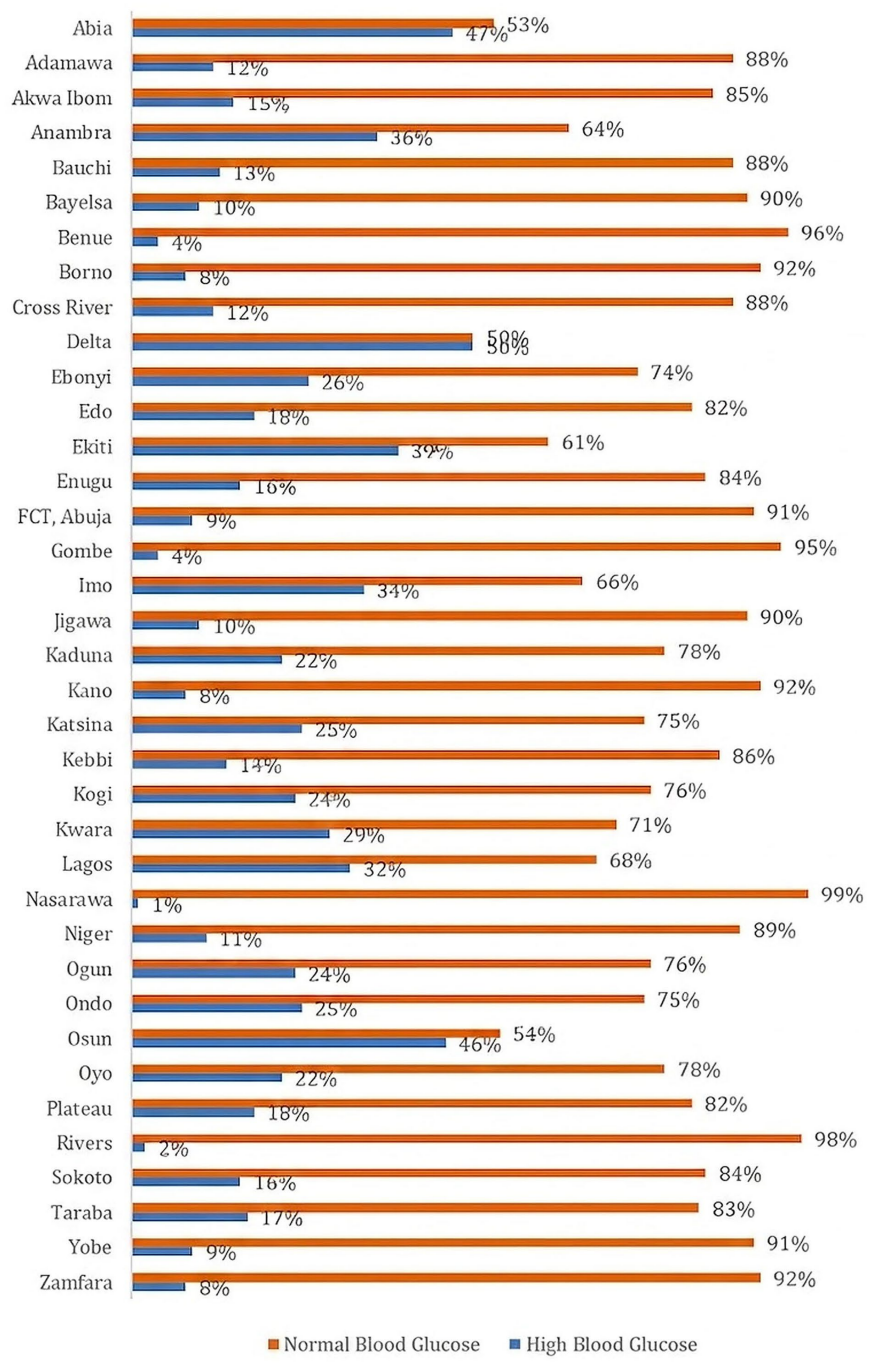
Blood glucose readings of screened participants categorized by state.

*Lowest burden states*: Nasarawa had the lowest readings at 1.4%, followed by Rivers (2.2%), Benue (4.2%), and Gombe (4.9%). Several other states, primarily in the North, showed rates below 10%, including Borno (8.3%), FCT (8.8%), Kano (8.1%), Yobe (9.5%), and Zamfara (7.8%) (Figure 10).

The vast range in high blood glucose proportions, from under 2 to 50%, strongly suggests highly localized factors influencing diabetes risk or detection across Nigeria. These factors require further investigation.

#### Lifestyle factors and NCD risk

The analysis explored associations between key lifestyle factors, smoking, alcohol consumption, and exercise, and high blood pressure and blood glucose (20). While some findings aligned with established evidence, others highlighted the complexity of behavioral determinants in NCD risk.

Overall, individuals who smoked (15%) or consumed alcohol (13%) recorded higher readings of high blood pressure than non-smokers and non-drinkers, while those who exercised showed slightly lower readings (12%). For high blood glucose, alcohol consumption was strongly associated with higher readings (29% versus 21%), whereas smoking showed no significant difference. Unexpectedly, individuals who reported exercising had higher blood glucose readings (24% versus 19%), suggesting possible reverse causation or confounding factors. These patterns reinforce that lifestyle–disease relationships are multifactorial and should be interpreted cautiously.

#### Association between lifestyle factors and high blood pressure

The analysis revealed statistically significant associations between three examined lifestyle factors (smoking, alcohol consumption, and exercise) and high blood pressure readings:

- *Smoking*: Participants who had ever smoked reported a higher proportion of high blood pressure (15%) compared with non-smokers (12%) (*p* < 0.001). This aligns with established evidence linking smoking to cardiovascular risk.
- *Alcohol consumption*: Those who reported alcohol use had a slightly higher proportion (13%) compared to non-drinkers (12%) (*p* < 0.001), suggesting a modest but statistically significant association.
- *Exercise*: Individuals who engaged in physical activity showed slightly lower readings of high blood pressure (12%) compared to those who did not (13%) (*p* = 0.007), suggesting a mild protective effect consistent with global evidence.

##### Association between lifestyle factors and high blood glucose

The relationship between lifestyle factors and high blood glucose readings presented some expected and some unexpected findings:

- *Smoking*: No significant association was observed between smoking history and high blood glucose (both 22%) (*p* = 0.13), indicating that smoking was not a substantial determinant within this dataset.
- *Alcohol consumption*: Participants who had ever consumed alcohol had a significantly higher proportion of high blood glucose (29%) than those who had not (21%) (*p* < 0.001), reinforcing known metabolic risks associated with alcohol use.
- *Exercise*: Individuals who reported exercising had higher blood glucose readings (24%) than those who did not (19%) (*p* < 0.001). This counterintuitive finding likely reflects reverse causation, whereby individuals already diagnosed with high blood glucose are more likely to initiate exercise. It may also indicate unmeasured variations in exercise type, duration, or intensity.

These results suggest that lifestyle factors influence NCD risk in complex ways, emphasizing the need for more detailed behavioral data and longitudinal research to establish causal pathways.

It is noteworthy that hypertension and type 2 diabetes mellitus frequently cluster as components of metabolic syndrome, alongside dyslipidaemia, central obesity, and insulin resistance (5). The campaign data did not permit a formal assessment of metabolic syndrome, as fasting lipid profiles and waist circumference were not captured. However, cross-tabulation of blood pressure and blood glucose outcomes in this dataset reveals that a proportion of participants exhibited elevations in both indicators simultaneously, pointing to the likely importance of shared cardiometabolic risk pathways. Future iterations of this campaign should consider collecting lipid profiles and waist circumference to enable a metabolic syndrome analysis.

#### Medication use among individuals with NCDs

The screening data provided essential insights into medication use among individuals who self-reported a diagnosis of hypertension across Nigeria.

#### Hypertension medication

Of 210,054 respondents, 71% (*n* = 148,674) reported currently taking antihypertensive medication. Among these, 80% (*n* = 119,115) recorded regular blood pressure readings at the time of screening, while 20% (*n* = 29,559) still had elevated readings. Among participants not on medication (*n* = 61,380), 21% (*n* = 12,791) had high blood pressure readings, and 79% (*n* = 48,589) had regular readings. These patterns indicate that while medication coverage among individuals who self-reported being hypertensive is high, treatment effectiveness and adherence may vary.

#### Diabetes medication

Of 88,615 respondents, 61% (*n* = 54,226) self-reported using glucose-lowering medication. Among this group, 77% (*n* = 41,955) had normal blood glucose levels, while 23% (*n* = 12,271) still recorded elevated readings. Among those not on medication (*n* = 34,389), 22% (*n* = 7,567) showed high blood glucose, and 78% (*n* = 26,822) had normal levels.

While these findings suggest moderate coverage of medication use, they also highlight possible gaps in diagnosis, continuity of care, and treatment adherence. Moreover, individuals not on medication but with “normal” readings could include those undiagnosed or recently improved through behavioral changes, potentially masking uncontrolled NCDs within the population.

### Comparative analysis with regional and national data

The Know Your Numbers; Control Your Numbers campaign recorded a 12% proportion of participants with high blood pressure readings, which is noticeably lower than national estimates of 22.5–28.9% for hypertension (21). Several factors could explain this difference, including variations in the types of individuals who participated, differences in measurement protocols, and the single-point nature of the readings, which may capture temporary elevations rather than clinically confirmed hypertension (17). It may also reflect improved public awareness or higher participation from individuals at lower cardiovascular risk.

In contrast, the 22% proportion of participants with high blood glucose observed during the campaign is substantially higher than Nigeria’s estimated 5.8% diabetes prevalence (WHO, 2024). This discrepancy is likely due to methodological factors such as the use of random (non-fasting) glucose testing, the absence of confirmatory assessments, and the possibility of stress- or meal-related temporary glucose elevations. However, it may also indicate a high proportion of undiagnosed or pre-diabetic individuals among those screened. Although these values are based on single-point screening measurements and are not diagnostic of hypertension or diabetes, they provide important early warning signals. They highlight the need for deeper investigation, especially as these indicators can influence the future burden of non-communicable diseases if left unchecked. The significant regional and state-level variations, particularly in high blood glucose, ranging from 9 to 47% across zones, demonstrate the limitations of relying solely on national averages for planning and resource allocation. High-burden states such as Delta, Abia, and Osun require further assessment to rule out measurement errors or sampling biases, while lower-burden states like Nasarawa and Rivers may reflect genuine demographic or contextual differences.

Several published Nigerian studies offer useful context for interpreting the campaign’s findings. The nationwide hypertension survey (8), which used confirmatory diagnostic criteria across a representative sample, estimated hypertension prevalence at approximately 38.1%, substantially higher than the 12% screening-positive rate observed in this campaign. This difference is expected, given the single-measurement, non-fasting, open-enrolment design of the Know Your Numbers campaign versus a population-representative study with standardized diagnostics.

Similarly (9), estimated national hypertension prevalence at 28.9% through systematic review, while (10) estimated a diabetes prevalence of approximately 5.8% in Nigeria (12). The campaign’s 22% high blood glucose rate substantially exceeds this estimate, likely due to the use of random blood glucose measurements rather than fasting glucose or HbA1c, and the potential inclusion of post-prandial transient elevations.

At the state level, the Abia State NCD Survey reported hypertension prevalence of approximately 32.5%, compared to the campaign’s 14% high blood pressure rate for Abia (11), a difference consistent with the methodological distinctions above. These comparisons reinforce that the campaign data represent screening signals and not diagnostic prevalence, and underscore the need for confirmatory studies to establish accurate state-level burden estimates.

These differences are likely driven by a combination of demographic factors, genetic predisposition, dietary and lifestyle patterns, socioeconomic conditions, environmental exposures, and variable access to screening and care. Differences in local implementation and device calibration may also have influenced results. Further investigation is therefore essential to verify these findings and better understand the underlying drivers of such disparities across Nigeria.

### Policy implications of findings

The Know Your Numbers; Control Your Numbers campaign (13) provides one of the most extensive real-world datasets on NCD risk factors in Nigeria, capturing population-level patterns across states and geopolitical zones. Beyond its scale, the dataset offers timely insights into emerging trends that are highly relevant for planning, prioritization, and resource allocation. Several strategic implications emerge from the findings for national and subnational policy.

- *Building a stronger evidence base for NCD planning*: The differences between the screening results and existing national estimates show how urgently Nigeria needs a more reliable system for tracking NCDs. A more coordinated approach would help the country standardize how blood pressure and glucose are measured, introduce confirmatory testing, and ensure states are collecting comparable data. This will give policymakers a clearer picture of the real burden and where to focus attention.
- Making NCD services part of routine PHC: The number of people with high blood pressure and blood glucose reinforces what the country has already recognized: NCD care needs to be part of everyday primary health care, not a separate program. This means PHCs routinely checking BP and glucose, offering brief counseling, strengthening referrals, and ensuring BHCPF-supported facilities can keep essential medicines available. This is central to Nigeria’s UHC journey.
- Focusing on equity and supporting states based on their actual needs: The wide differences across states show that a “one-size-fits-all” approach will not work. States in the North-West and South-East, for example, may need more targeted support due to higher metabolic risks. Using platforms like NHCF to guide states based on their specific burden will help ensure resources reach the places that need them most (15).
- Tackling lifestyle and environmental factors that drive NCDs: The connection between lifestyle behaviors and NCD risk means Nigeria cannot address NCDs through the health sector alone. Policies that promote healthier diets, regulate tobacco and alcohol, improve walkability and safe public spaces, and strengthen community health promotion will make a significant difference. This aligns with the country’s multi-sectoral NCD action plan and national development goals.
- Taking a closer look at outlier states: Some states—such as Delta, Abia, and Nasarawa reported extremely high or very low blood glucose proportions. These results need follow-up to understand whether they reflect real community patterns or issues with sampling or measurement. State-led reviews will help ensure planning and budgeting reflect the true situation on the ground.
- Ensuring sustainable financing and clearer national coordination: NCD prevention and control will only improve if there is consistent domestic funding and stronger coordination between federal and state actors. Integrating NCD priorities into the country’s health security framework will help Nigeria reduce reliance on donor-funded, fragmented efforts and create a more sustainable system.

## Lessons learned, limitations, and recommendations

### Lessons learned

The nationwide implementation of Know Your Numbers, Control Your Numbers offers several critical lessons for future public health programming in Nigeria:

1. *Scale and reach*: The project demonstrated the feasibility of conducting large-scale health screening initiatives across Nigeria’s diverse landscape, providing a model for future national health programs.
2. *Importance of granular data*: The significant variations observed across states and geopolitical zones underscore the critical need to collect and analyze geographically disaggregated data to move beyond national averages and implement truly targeted interventions.
3. *Demographic insights*: Consistent patterns by age and gender highlight the need to incorporate demographic factors into the design of all NCD prevention and control programs.
4. *Data system value*: The project emphasizes the importance of standardized data collection and robust data management systems to generate reliable evidence to inform national health policy.
5. *Need for comprehensive data*: The absence of lifestyle and medication data in the current analysis highlights a limitation and underscores the need to capture a broader range of variables in future NCD surveillance and research to understand the epidemic and its response fully.

### Policy and programmatic recommendations

To translate insights from the Know Your Numbers; Control Your Numbers campaign into sustained progress on noncommunicable disease (NCD) control, the following priority actions are recommended for the Federal Ministry of Health & Social Welfare (FMoH), the Nigeria Health Commissioners’ Forum (NHCF), and State Ministries of Health (SMoHs):

1. *Institutionalize state-led NCD action plans under the national strategic framework (2023–2027)*: Support each state to develop and operationalize a costed NCD Action Plan aligned with the National Strategic Plan for NCD Prevention and Control (2023–2027) (14). High-burden states such as Delta, Abia, Kwara, and Plateau should serve as demonstration sites, piloting scalable, community-based interventions through existing PHC structures.
2. *Integrate NCD services into the basic health care provision fund (BHCPF)*: Expand the BHCPF service package to include screening, counseling, and follow-up for hypertension at PHCs. This should be complemented by results-based financing mechanisms that reward LGAs and PHCs for achieving screening and referral targets.
3. *Embed NCD tasks into the CHIPS program and ward development committees*: Equip Community Health Influencers, Promoters, and Services (CHIPS) agents and Ward Development Committees to conduct routine community NCD screening, referrals, and awareness drives. This will extend NCD prevention into rural areas and strengthen community ownership in line with the Primary Health Care under One Roof (PHCUOR) policy.
4. *Deploy a national “Know Your Numbers” digital monitoring dashboard*: Establish a real-time digital platform to track NCD screening, referrals, and treatment data from PHCs and state programs. The platform linked to the National Health Management Information System (NHMIS) and NPHCDA dashboards should support evidence-based decision-making, early detection of data anomalies, and accountability for results.
5. *Pilot an NCD task-shifting and task-sharing model for PHC workers*: Approve and implement standardized protocols that empower trained community health extension workers (CHEWs) and nurses to conduct blood pressure and glucose management (screening, counseling, and medication titration) under clinical supervision. This approach builds on Nigeria’s success with HIV and RMNCH task-shifting and helps bridge the human resource gap for NCD care.
6. *Launch the “Healthy Habits Naija” national behavior change campaign*: In collaboration with the Federal Ministries of Education, Youth, and Information, roll out a sustained multi-platform campaign promoting healthy diets, regular physical activity, and reduced tobacco and alcohol consumption. The campaign should harness influencers, schools, and workplaces to normalize healthier behaviors among youth and adults.
7. *Establish a national NCD medicines and diagnostics access compact*: Through collaboration between the National Health Insurance Authority (NHIA), FMoH, and State Health Insurance Schemes, introduce pooled procurement and price negotiation mechanisms to ensure affordable access to essential NCD drugs and diagnostic devices at the PHC level, mirroring the success of pooled vaccine procurement systems.
8. *Allocate dedicated NCD budget lines in state health plans*: Mandate that each state include specific NCD allocations within its annual health budget and operational plans. Funding should cover routine screening equipment, referral systems, and health worker training, with performance tracked through measurable indicators at the LGA level.
9. *Commission a national validation study*: Conduct a follow-up national validation study using standardized confirmatory testing (fasting glucose, repeat BP) to refine burden estimates through the NHCF to promote cross-state sharing of innovations in screening, digital health, and financing mechanisms.
10. *Mainstream NCD control within the health security and UHC agendas*: Position NCD prevention as a key pillar of Nigeria’s Health Security Architecture and Universal Health Coverage roadmap. By embedding NCD screening, referral, and data systems into emergency preparedness, maternal health, and routine PHC services, Nigeria can build a more resilient, integrated health system.

### Limitations and challenges

The findings and recommendations presented in the Nigeria Know Your Numbers; Control Your Numbers should be considered, considering several potential limitations and challenges inherent in large-scale screening initiatives:

1. *Sampling considerations*: Participation in the screening was voluntary. Although the overall sample size was large (1,551,394), the self-selected nature of enrolment may have introduced selection bias, with over-representation of individuals who are more health-conscious or who have easier access to screening sites. This may result in either an under-estimate (if healthier individuals predominate) or an over-estimate (if those already concerned about their health are more likely to attend) of the true population burden. Furthermore, states with relatively small sample sizes; including Zamfara (*n* = 64), Taraba (*n* = 42), Katsina (*n* = 138), Sokoto (*n* = 86), and Kebbi (*n* = 81); are not adequately representative of their state populations and findings for these states should be interpreted with particular caution.
2. *Data quality variations*: Potential variations in data collection quality, adherence to protocols, and equipment calibration across numerous screening sites and states could introduce variability into the aggregated data.
3. *Cross-sectional nature*: The screening data provide a snapshot of potential NCD prevalence at a specific point in time and do not capture longitudinal trends, disease progression, or the impact of interventions over time.
4. *Case definition*: Classification of high blood pressure and high blood glucose was based on single screening measurements, which may differ from formal clinical diagnostic criteria that require multiple assessments.
5. *Self-reported data*: Information on lifestyle factors (smoking, alcohol, exercise) and medication history was collected via self-report, which may be subject to recall or social desirability bias.
6. *Missing data*: Incomplete data for certain variables or participants, potentially due to logistical challenges or participant refusal, could affect the completeness and representativeness of some analyses (e.g., medication adherence).
7. *Single-measurement diagnostic limitation*: Blood pressure was measured once per participant without a rest or repeat reading, and blood glucose was assessed as random (non-fasting) values. Both measurements therefore fail to meet standard diagnostic criteria and may over- or underestimate true prevalence. These findings should be considered screening signals only.
8. *Absence of urban–rural stratification*: Although screening took place at both fixed health facilities and mobile outreach sites, no systematic distinction was made between urban and rural recruitment points. Urban sites may attract a disproportionately health-aware population, limiting generalisability to rural communities.
9. *Multi-morbidity not assessed*: No attempt was made to assess the co-occurrence of hypertension and diabetes within the same individual, nor their clustering as components of metabolic syndrome. This limits the interpretability of associations between the two conditions.
10. *BMI and anthropometric*: Although anthropometric measures (height and weight) were collected, body mass index was not incorporated into the analysis. Given the established relationship between adiposity and both hypertension and type 2 diabetes, this omission limits the epidemiological depth of the findings.
11. *Duration and multi-morbidity of prior diagnoses unknown*: Among participants who self-reported a prior diagnosis of hypertension or diabetes, duration of diagnosis and the presence of co-existing conditions were not captured or reported. This prevents any assessment of disease chronicity or treatment adequacy over time.

Despite these limitations, the Know Your Numbers; Control Your Numbers data provide invaluable insights into the national burden and distribution of risk factors for hypertension and diabetes in Nigeria, establishing a crucial evidence base for policy and future research.

## Conclusion

*Know Your Numbers; Control Your Numbers* represents a landmark national effort to understand and address the burden of high blood pressure and blood glucose across Nigeria. The campaign has provided valuable, large-scale evidence on the distribution of NCD risk factors, revealing significant demographic and geographic variations that demand nuanced, locally informed responses. While the findings are not diagnostic, they highlight an urgent need for sustained investment in NCD prevention, screening, and management anchored within the primary healthcare system. Tackling these challenges will require strong political leadership, improved health financing, reliable data systems, and multi-sectoral collaboration that links health with education, urban planning, and social policy.

By translating these insights into coordinated action, Nigeria can advance toward Universal Health Coverage, achieve its SDG 3.4 targets on reducing premature NCD mortality, and build a healthier, more productive population for the future.

## Data Availability

The raw data supporting the conclusions of this article will be made available by the authors, without undue reservation.
