# Correction: Know Your Number; Control Your Number campaign: Nigeria

**DOI:** 10.3389/fpubh.2026.1883571

**Published:** 2026-06-03

**Authors:** Nigeria Health Commissioners' Forum (NHCF), Ayotunde Oguntade, Emmanuella Ezike, Ahmed Akorede Zakarriyah, Oladapo Awobeku

**Affiliations:** 1 Nigeria Health Commissioners Forum, Abuja, Nigeria; 2 Isuna Technologies, Abuja, Nigeria

**Keywords:** high blood glucose and blood pressure, Know Your Numbers, Nigeria, noncommunicable diseases, public health surveillance, screening

The order of authors in the author list of the published paper was erroneously written as:

Ayotunde Oguntade^2^, Emmanuella Ezike^2^^*^, Ahmed Akorede Zakarriyah^2^, Oladapo Awobeku^2^, Nigeria Health Commissioners' Forum (NHCF)^1^

The correct author list reads:

Nigeria Health Commissioners' Forum (NHCF)^1^, Ayotunde Oguntade^2^, Emmanuella Ezike^2^^*^, Ahmed Akorede Zakarriyah^2^ and Oladapo Awobeku^2^

^1^ Nigeria Health Commissioners Forum, Abuja, Nigeria

^2^ Isuna Technologies, Abuja, Nigeria

The quality of Figures 1–10 was unclear and difficult to read.

**Figure 1:**
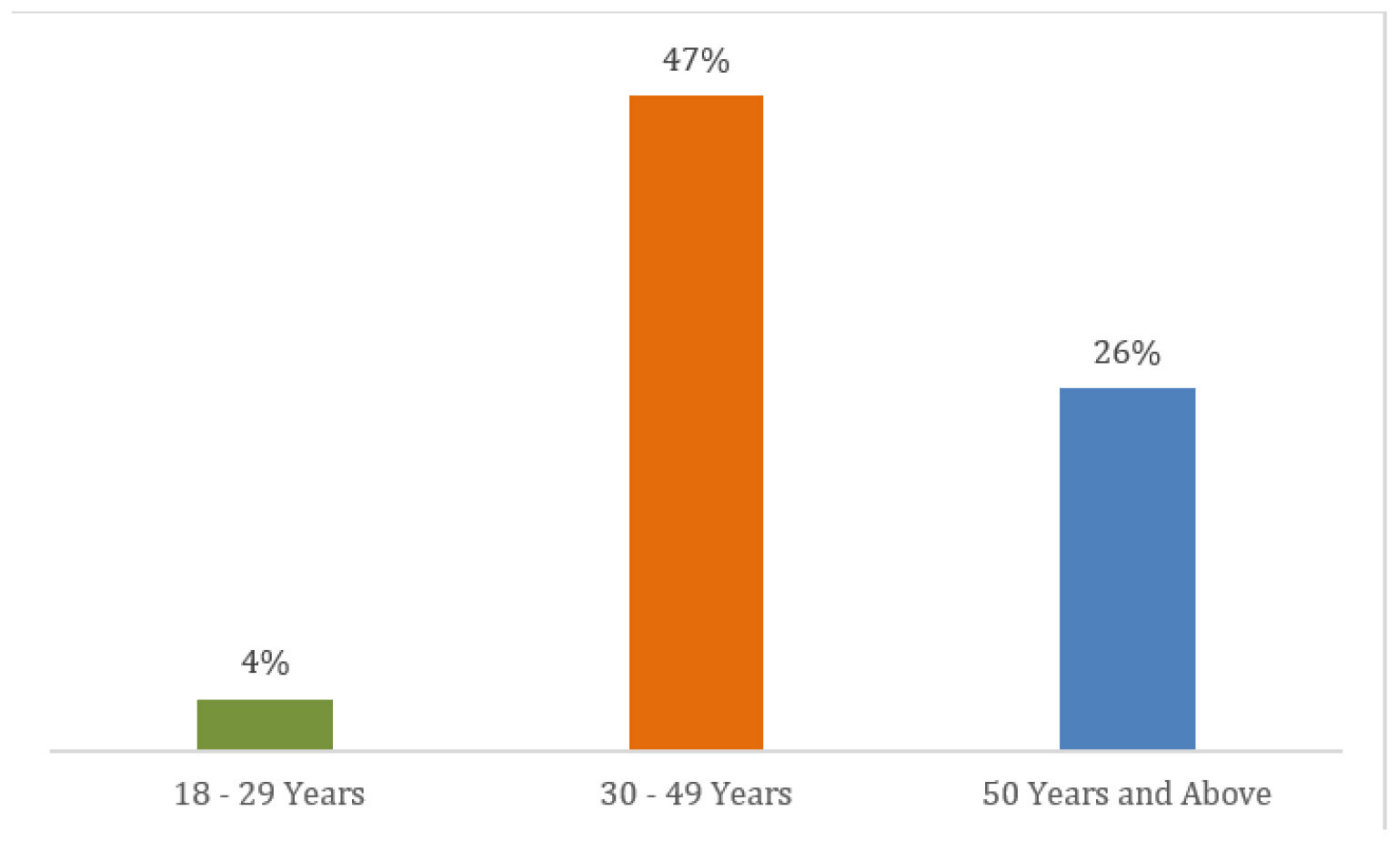
Age distribution of screened participants *N* = 1,551,393.

**Figure 2:**
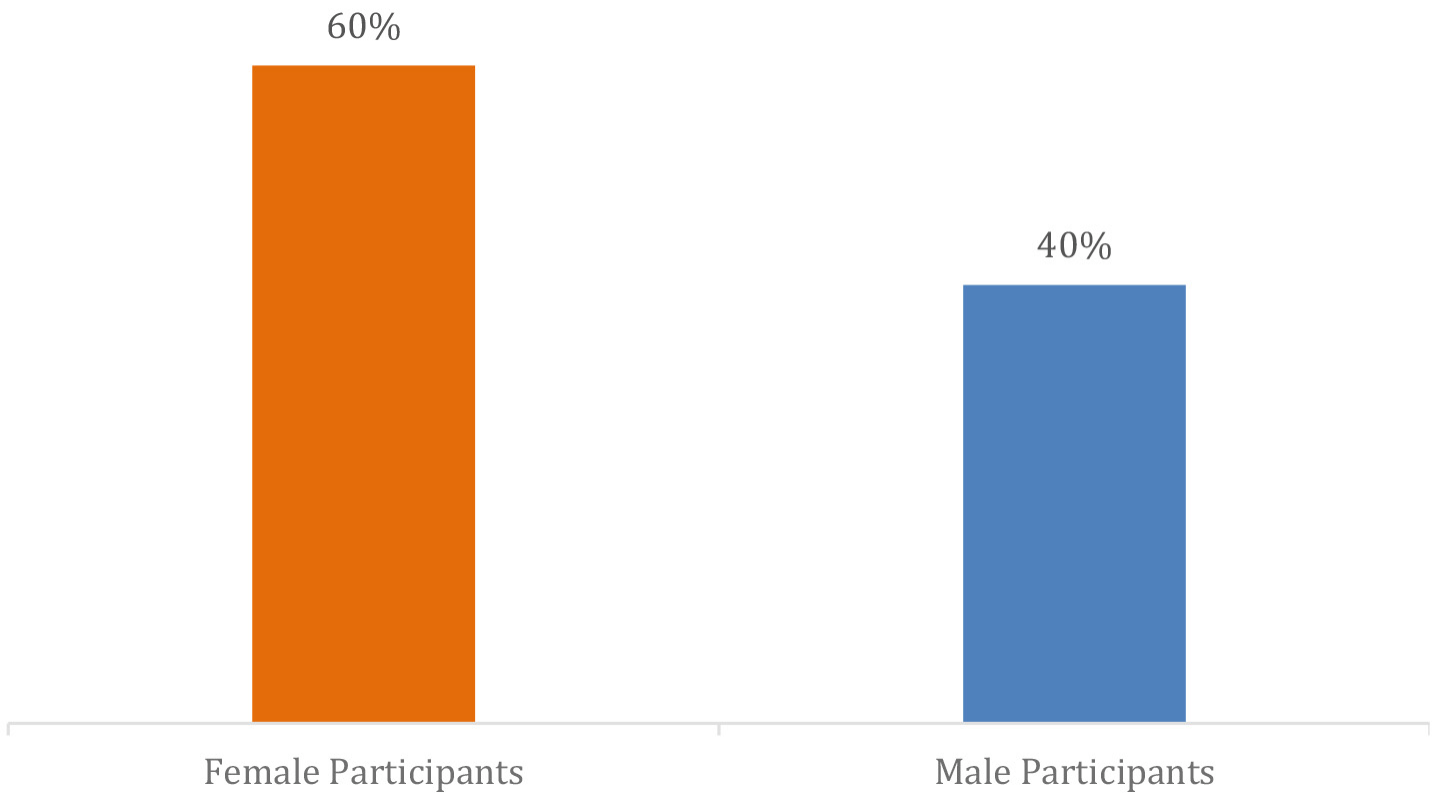
Gender distribution of screened participants *N* = 1,551,393.

**Figure 3:**
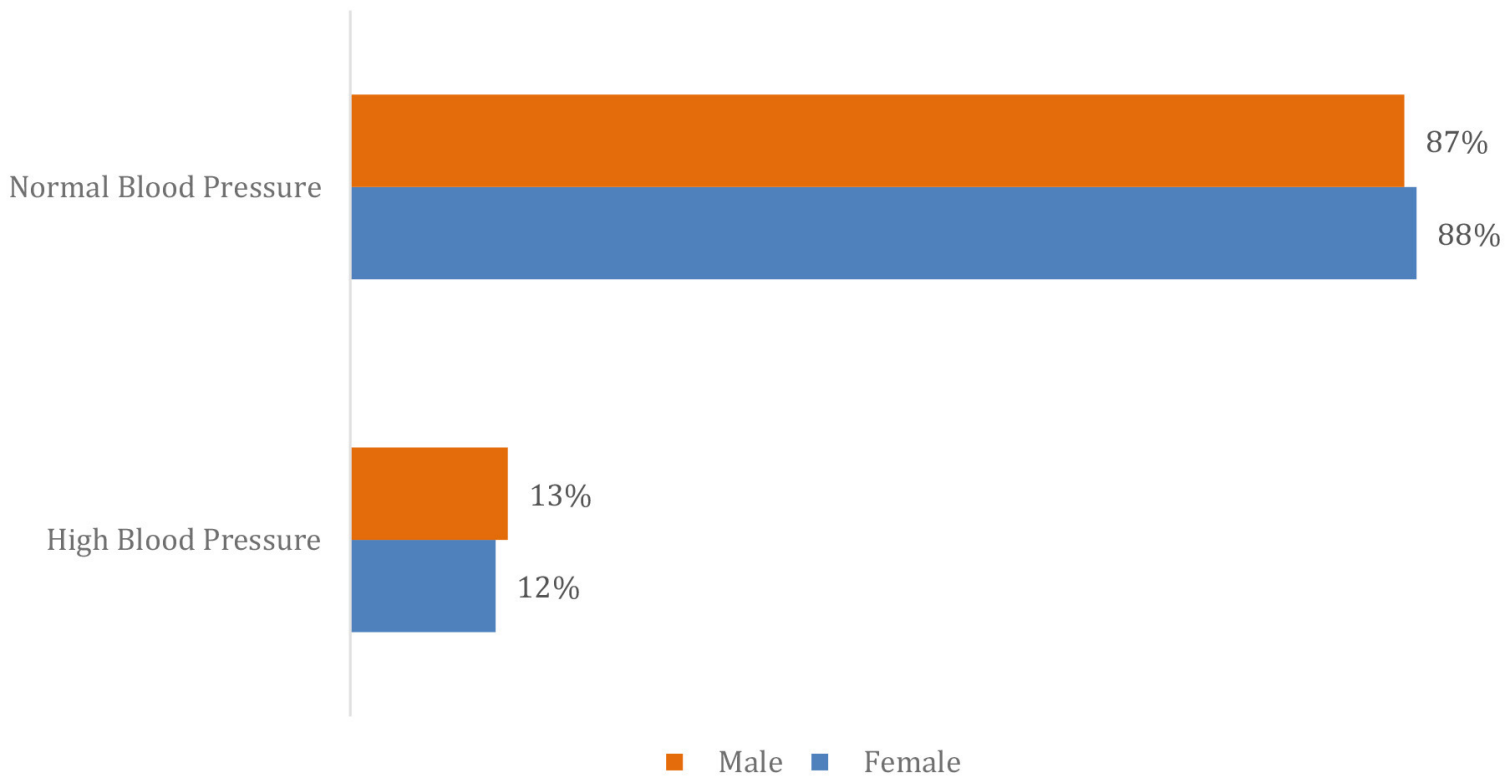
Blood pressure readings of screened participants categorized by gender.

**Figure 4:**
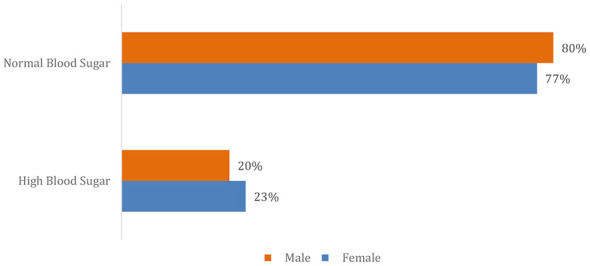
Blood glucose readings of screened participants categorized by gender.

**Figure 5:**
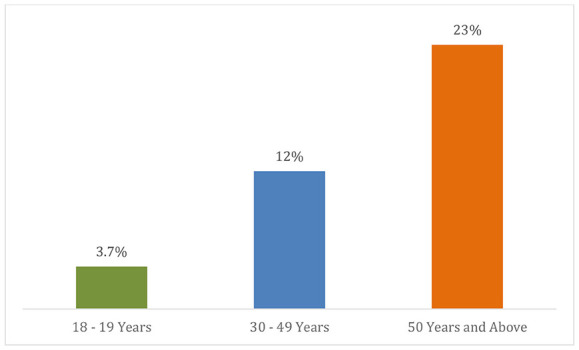
Proportion of high blood pressure in age-related patterns.

**Figure 6:**
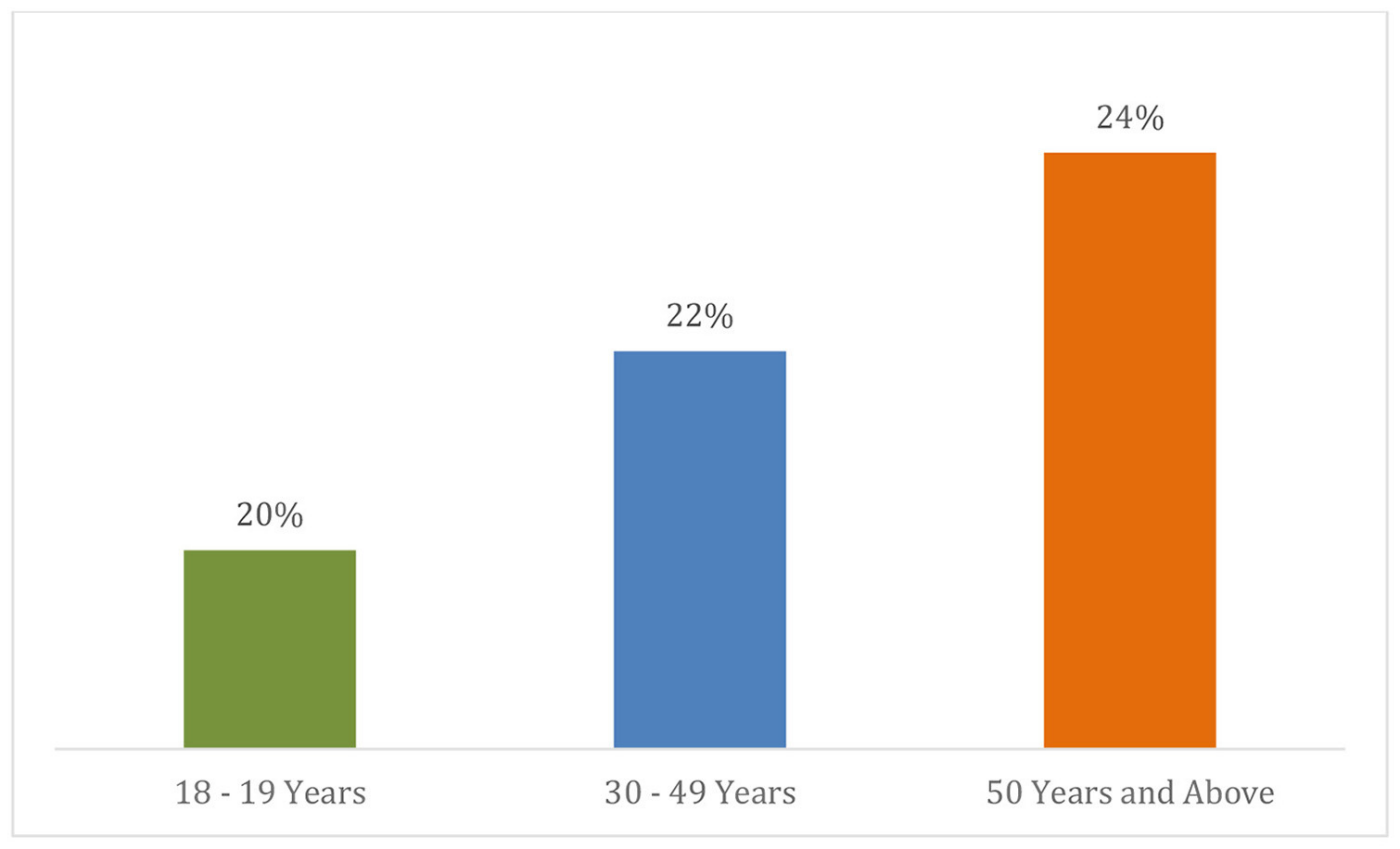
Proportion of high blood glucose in age-related patterns.

**Figure 7:**
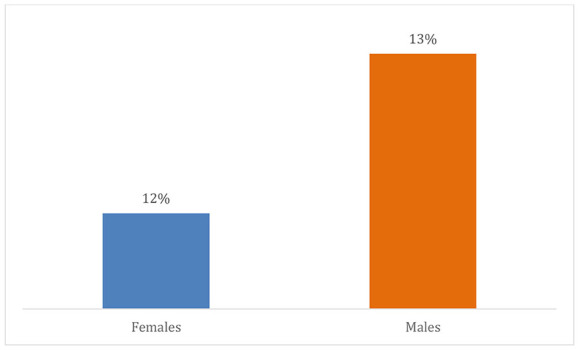
Prevalence of high blood pressure in gender-related patterns.

**Figure 8:**
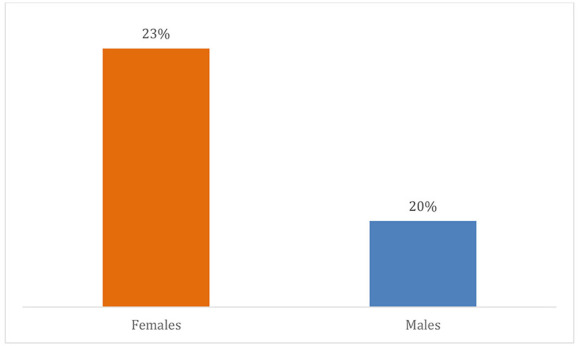
Prevalence of high blood glucose in gender-related patterns.

**Figure 9:**
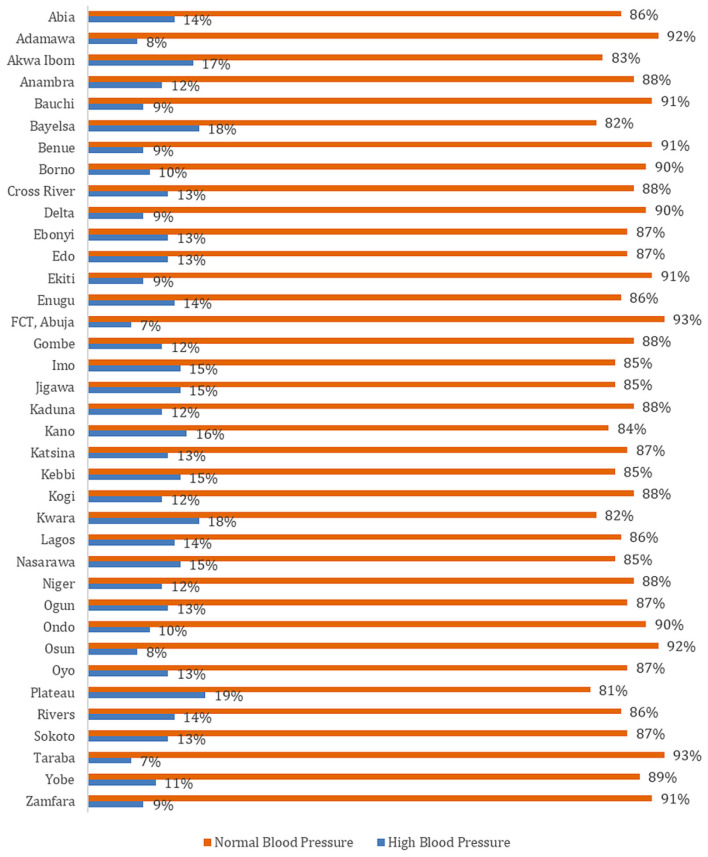
Blood pressure readings of screened participants categorized by state.

**Figure 10:**
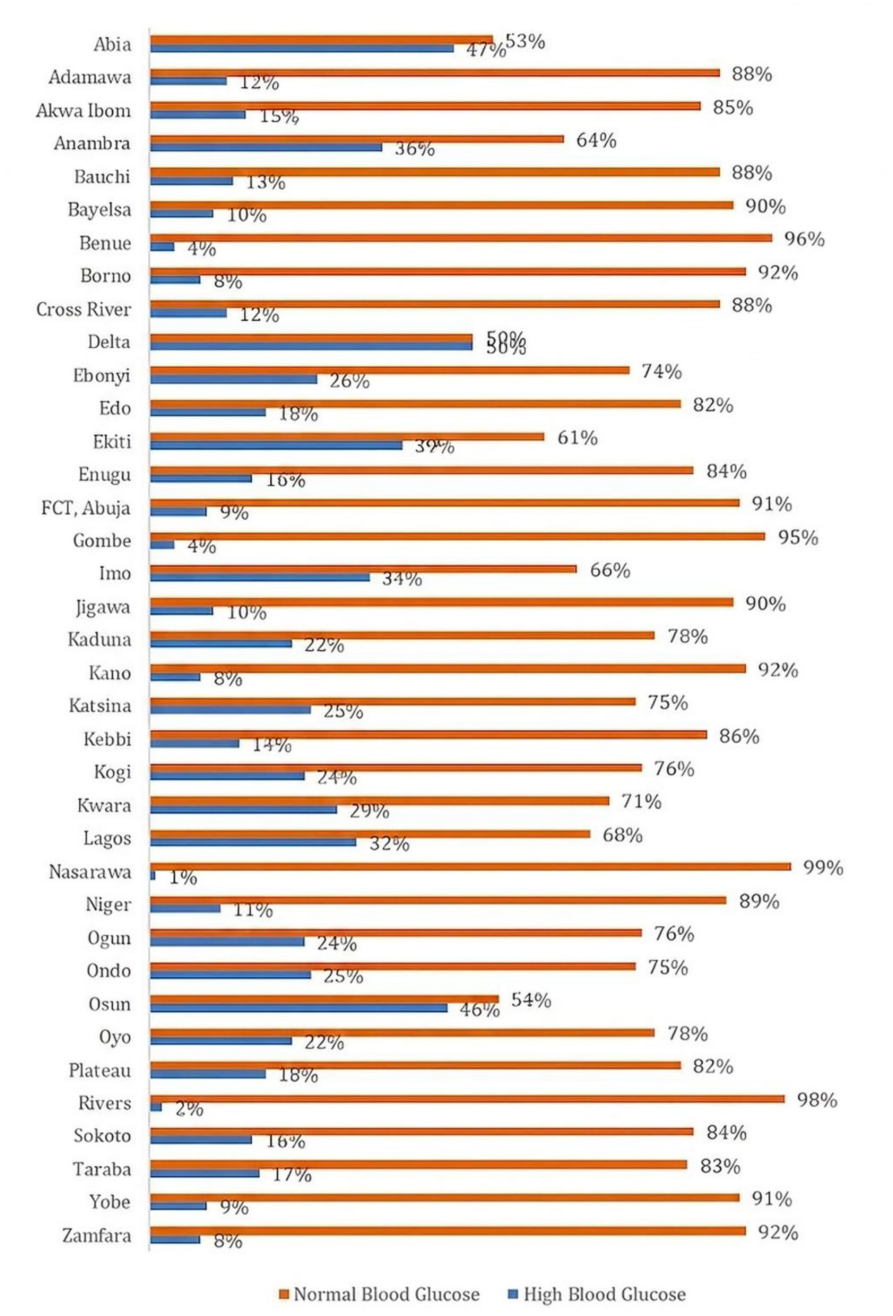
Blood glucose readings of screened participants categorized by state.

The corrected version of Figures 1–10, with a higher image resolution and quality, appears below

The original version of this article has been updated.

